# What Makes a Robot Social? A Review of Social Robots from Science Fiction to a Home or Hospital Near You

**DOI:** 10.1007/s43154-020-00035-0

**Published:** 2021-02-04

**Authors:** Anna Henschel, Guy Laban, Emily S. Cross

**Affiliations:** 1 Institute of Neuroscience and Psychology, Department of Psychology, University of Glasgow, Glasgow, Scotland; 2 Department of Cognitive Science, Macquarie University, Sydney, Australia

**Keywords:** Social robots, Human-robot interaction, Socially assistive robots, Cognitive neuroscience, Social cognition

## Abstract

**Purpose of Review:**

We provide an outlook on the definitions, laboratory research, and applications of social robots, with an aim to understand what makes a robot social—in the eyes of science and the general public.

**Recent Findings:**

Social robots demonstrate their potential when deployed within contexts appropriate to their form and functions. Some examples include companions for the elderly and cognitively impaired individuals, robots within educational settings, and as tools to support cognitive and behavioural change interventions.

**Summary:**

Science fiction has inspired us to conceive of a future with autonomous robots helping with every aspect of our daily lives, although the robots we are familiar with through film and literature remain a vision of the distant future. While there are still miles to go before robots become a regular feature within our social spaces, rapid progress in social robotics research, aided by the social sciences, is helping to move us closer to this reality.

## Introduction

Since its inception, the scientific field of robotics has been closely entwined with the science fiction literature, with the first mention of the word robot made by Karel Čapek in his 1920 play ‘Rossum’s Universal Robots’. In this play, robots who look almost indistinguishable from humans are exploited as factory slaves and later rebel against their human makers, a popular trope in science fiction. A bit later, the term ‘robotics’ was coined by Isaac Asimov in his 1941 short story ‘Liar!’, which features a robot that is compelled to lie so as not to upset its human creators. While these terms were introduced historically quite late, visions of automata have existed for almost as long as humans have lived together in societies. Spanning back to at least ancient Egypt, Greece, and China, and including the Golem from Jewish mythology, the eighteenth century ‘Turk’ (a fake chess playing machine, controlled by a human hiding inside the device) and the friendly Japanese ‘Gakutensoku’ mechatronic puppets and automatons have fuelled the public imagination across cultures and history, in terms of what might be possible in terms of human-fabricated autonomous agents that interact with us—almost as equals [1, 2].

**Fig. 1:**
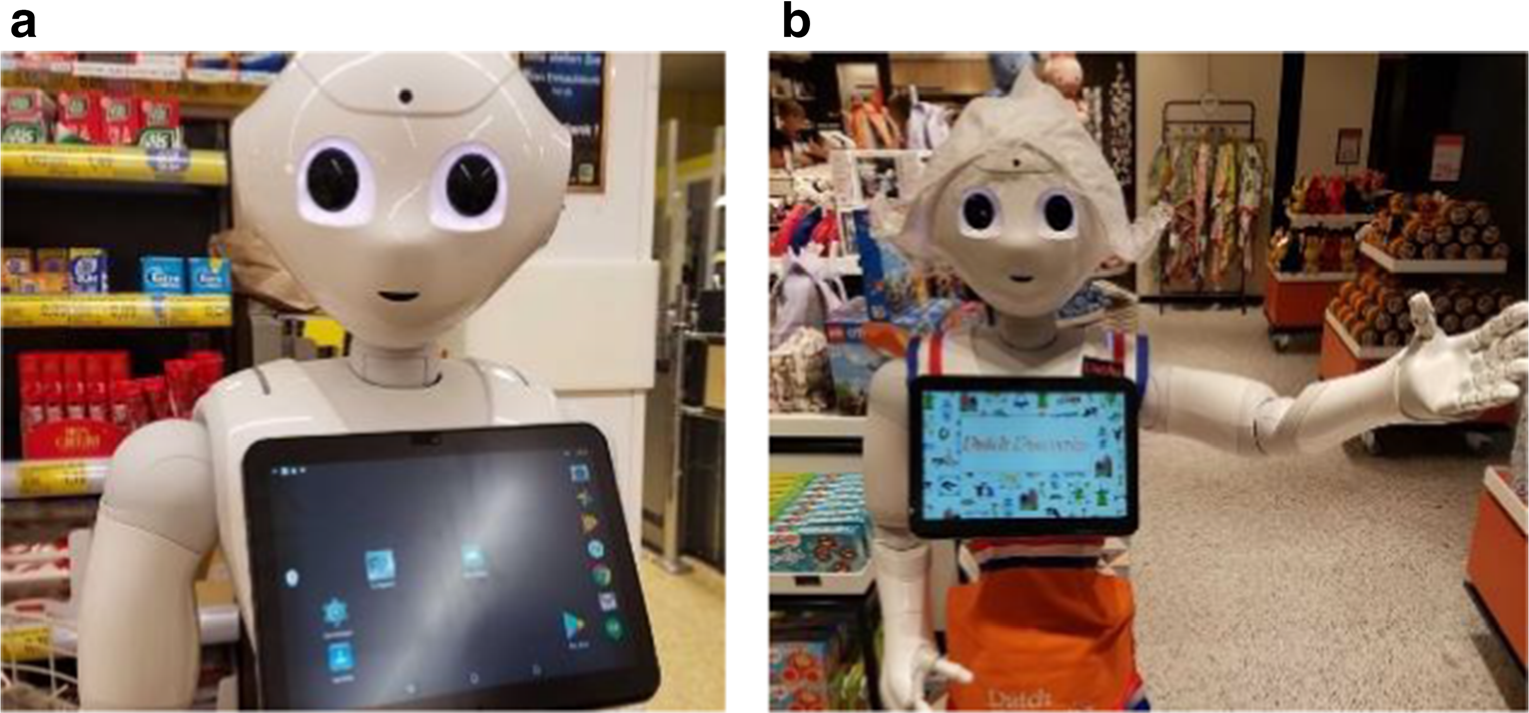
Recent examples of the Pepper robot ‘in the wild’. **a** The social robot was placed at the customer checkout in a German supermarket and reminded shoppers of new hygiene regulations to ensure public health in April 2020, during the global coronavirus pandemic. **b** Pepper in a Dutch souvenir shop at Schiphol airport. (Photos taken by Anna Henschel)

Science fiction has further inspired us to conceive of a future where autonomous robots help with every aspect of our daily lives, although the robots we are familiar with through films like *Ex Machina* or *Robot & Frank* remain a vision of the distant future, whether they are depicted as helpers and companions, or villains [3••]. When we encounter robots ‘in the wild’ (Fig. 1), this discrepancy between the reality of social robots and our expectations towards them becomes even more salient. Accordingly, Duffy and Joue coined the ‘social robot paradox’, which has remained a critical point in social robotics over the years [4]. Speaking of this paradox, Duffy states:

In fact, humanoid robots outside of science fiction, have thus far only been toys or research platforms with nebulous applications. It is intriguing that one of the most powerful paradigms for adaptivity and flexibility, the human, has so far, when modelled in the form of a machine, resulted in little more than a toy. Its usefulness is very limited. (p. 1)

Many social robot developers have designed their creations to incorporate human characteristics, while at the same time being careful to avoid imitating human appearance or motion too closely, in order to avoid falling into the Uncanny Valley [5]. While a human-like embodiment as a design feature for social robots is a powerful signal to users that the agent affords social interactions, it also makes the robot more prone to failing to deliver on high expectations regarding the nature of the interaction (e.g. [6–8]).

**Fig. 2:**
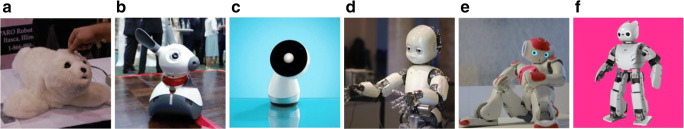
Examples of several social robotics platforms that are heavily used in research and/or have enjoyed commercial success, and are discussed in this review. **a** Paro, the cuddly baby harp seal robot. **b** MiRo, the puppy/bunny-like robot. **c** Jibo, the erstwhile personal home assistant robot. **d** iCub, the humanoid robot testbed for human cognition and AI. **e** Nao, a humanoid robot. **f** Darwin, a small humanoid robot (now discontinued)

This observation still rings true, with new social robots moving away from referencing the human form. Zoomorphic and pet-like robots (e.g. the Paro and MiRo robots, see Fig. 2) have been developed to enter peoples’ homes and address specific needs of their target populations (e.g. within care settings, with older adults, and with people with cognitive impairment). One way in which social robots and other kinds of artificial agents can provide acceptable solutions to people’s social needs (in certain situations) is by not raising peoples’ expectations of their capabilities to unrealistic levels. The importance of setting people’s expectations to appropriate levels is highlighted by the robot Jibo (Fig. 2), which also serves as a cautionary tale of this point. Jibo was among the first social robots developed for private consumers and was introduced in 2014 as a family robot designed to take up residence in people’s homes, to establish social relationships with them and serve as a personal assistant [9, 10]. By 2017, the company announced layoffs [11], sold their intellectual property and assets in 2018 [12], and by 2019, Jibo announced to its users the imminent shutdown of its servers [13].

While Jibo ultimately failed, disembodied and functional personal assistants like Amazon Alexa or Google Assistant which neither reference the human form nor are designed to establish social relationship with users, have been commercially successful [14–17]. Following on from Duffy and Joue’s suggestion [4], it could be that attempts to create ever more human-like robots, in terms of form and function, leads to unrealistic expectations of robots’ capabilities in human users, and thus, less effective human-robot interactions. Instead of trying to design social robots in line with science fiction’s unrealistic expectations, it will be important to understand when and why a robot should look or behave in a human-like way, and when this approach is ineffective or problematic. This observation raises questions regarding the value and definitions of what the concept of ‘social’ means within the interdisciplinary field of human-robot interaction.

In the current review, we provide an outlook on the definitions, laboratory research, and application of social robots. We begin by examining definitions of a social robot through the eyes of both scientists and users. Next, we address the lack of social and behavioural science research in social robotics, what the field can learn from social, behavioural, and neurocognitive research, and how principles from these disciplines are applied in today’s current social robots. Finally, we review some of the areas of social robots’ application that successfully capitalize upon robots’ social design and abilities.

## What Is ‘Social’ About Social Robots?

In the social robotics literature, no universally agreed-upon definition for social robots exists. Furthermore, consensus is lacking in terms of understanding what these robots do and what, specifically, makes them social. Within the field of HRI, social robots take on a special role, and fall under the category of ‘proximate interaction’, in which ‘humans and robots interact as peers or companions’ [18]. Based on reference information of articles they extracted, Mejia and Kajikawa [19] identified relevant clusters that represent the social robotics knowledgebase. The largest clusters in social robotics research can be summarized as ‘robots as social partners’ and ‘human factors and ergonomics in human-robot interaction’. Interestingly, the authors point out that research trends emphasize the various fields of application for social robots: robots as companions, robots as educators for children, and robots as assistants for the elderly. This is consistent with a trend identified by Šabanović, who, in interviews with robotics researchers in the USA and Japan, identified that social robots ‘often represent technological fixes’ i.e. using a technological approach to solve a pressing societal problem ([20], p. 349).

Sarrica and colleagues [21] investigated the question of how social robots are understood by analyzing definitions in articles published by the International Journal of Social Robotics between 2009 and 2015. In investigating the most often cited definitions, it becomes apparent how heterogenous the understanding of social robots is. Through this work, Sarrica and colleagues identified a few shared traits: social robots are physically embodied agents that have some (or full) autonomy and engage in social interactions with humans, by communicating, cooperating, and making decisions. These behaviours are then interpreted by human onlookers as ‘social’, according to current norms and conventions.

A study by de Graaf, Allouch, and van Dijk [6] evaluated users’ perspectives on the characteristics of social HRI through a longitudinal home study. They observed and identified eight main social characteristics that users described as factors for a social robot to appear as social and be accepted as social entities in their homes. The most prominent factor was (1) the capability of *two-way interaction*, expecting a robot to be able to respond to a human in a social manner. When a robot failed to do so, people were disappointed and experienced a sense of dissonance. Following this, users described the need for robots to share the same environment as them (be physically embodied or embedded), and to: (2) display *thoughts and feelings*; (3) be *socially aware* of their environment; (4) provide *social support* by being there for them (like their friends); and (5) demonstrate *autonomy*. Participants also raised the concepts of (6) *cosiness*, (7) *similarity to self*; and (8) *mutual respect*. However, these latter three concepts were mentioned fewer times than the previous five concepts. While users’ perceptions of robots’ socialness share many similarities with scholars’ definitions of social robots, some key differences also emerge. Users’ expectations, as described in de Graaf and colleagues’ [6] study, were influenced by their relationships with other social actors (i.e. their friends). Participants repeatedly compared the robot in that study to their friends, dwelling on the fact that the robot’s lack of social capabilities meant that it would be unlikely to become an actual ‘friend’. By contrast, the definitions of a social robot described in Sarrica and colleagues’ [21] review focus on general social and communication capabilities. It is of note, however, that these definitions rarely address the context of the interaction, whose importance is underscored by the findings of de Graaf and colleagues [6].

This discrepancy has been noted in other user studies as well. Dautenhahn and colleagues [22] show that participants in their studies did not see robots as companions or friends, but rather as useful household servants. Dereshev and colleagues [7] interviewed long-term, expert users of the Pepper robot (SoftBank Robotics; seen in Fig. 1). Their participants had lived and interacted with the robot on timescales ranging between 8 months to more than 3 years. The researchers report that one specific expectation regarding the humanoid Pepper robot was its ability to engage in a reciprocal conversation. Participants were disappointed when the robot was not able to go beyond the smart-speaker like single-turn structure of conversation. One of the participants also pointed out that people who interacted with Pepper quickly lost interest, a finding which is echoed in a usability study by Aldebaran (later purchased by SoftBank Robotics), where Pepper was deployed to the homes of users over several weeks [8]. The novelty effect is a common problem in social robotics, and long-term studies have often found a reduced engagement with various robotic platforms over time [23, 24].

Finally, Baraka and colleagues [25] recently proposed an ‘extended framework’ for social robotics by illustrating seven relevant dimensions of social robots: a robot’s (1) appearance, (2) social capabilities, (3) autonomy, (4) intelligence, the (5) proximity and (6) temporal profile of the interaction, and the (7) context of the interaction (such as its purpose or intended application). In their appearance classification system, they distinguish between bio-inspired robots (e.g. human- or animal-inspired), artefact shaped (e.g. robots resembling man-made objects or those that are imaginary), and functional robots (e.g. drones). Additional recent efforts to establish frameworks for designing and evaluating social robotics research emphasize that in all the enthusiasm from researchers from different fields to amplify or focus on social aspects of social robots, these robots remain, at their core, machines, and advances in HRI research will be well served to keep robots’ machine or object-like qualities in mind as well [26].

## Interdisciplinary Tensions

**Fig. 3:**
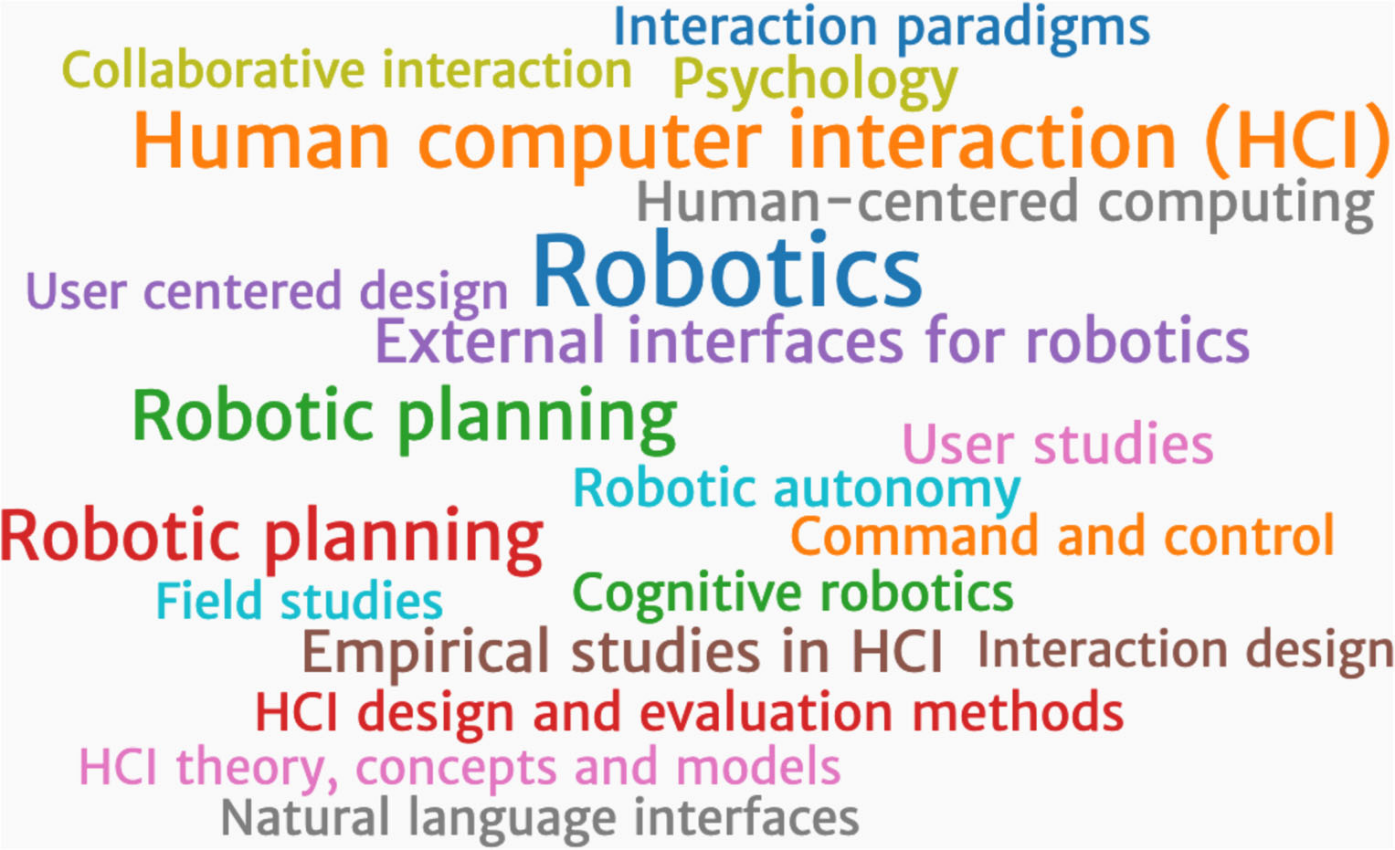
Subject areas in the ACM-HRI conference proceedings. The subject areas are presented as a word cloud with the size of the word representing the number of conference proceedings in one category. Robotics being the most frequent one (947 results), there are some nods to the social sciences: psychology (143 search results), user studies (175 results), and empirical studies in interaction design (57 results). (Screenshot taken from https://dl.acm.org/conference/hri)

The bibliometric analysis by Mejia and Kajikawa [19] referenced above also highlights that the social robotics literature comprises only a small portion (2.3%) of the larger robotics knowledgebase. When further investigating the extant social robotics literature, Mejia and Kajikawa [19] find that even though concepts of socialness play a central role, the social sciences are hardly represented. The authors write aptly: “Social robotics is social in its intention, but its knowledgebase is concentrated in the engineering and technology domains” (p.11). This lack of social, behavioural, and cognitive science input into social robot development highlights a challenge and an opportunity for future roboticists to work towards effective interdisciplinary collaborations with social scientists. Indeed, while the interdisciplinary nature of social robotics is emphasized throughout the literature, this observation by Mejia and Kajikawa reveals an interesting tension that has also been voiced by Broadbent [3••] and Eyssel [27]—the literature could benefit from knowledge about the mechanisms of human social behaviour gained through psychology, cognitive science, and neuroscience. As Fig. 3 illustrates, texts gathered from the proceedings from one of the premiere conferences debuting new empirical and theoretical work in social robotics (ACM-HRI) include some social science mentions, even if these concepts are not among this conference’s (current) core content. Irfan and colleagues [28] argue that as HRI is positioned between engineering and the social sciences (specifically social and cognitive psychology), HRI researchers should aim to develop novel methodology inspired by these scientific disciplines, while also learning from the mistakes and successes of these fields. With psychology researchers continuing to grapple with the replication crisis (referring to the concerning lack of reproducibility of published findings), HRI researchers would be well served to keep in mind these new approaches and methods to ensure their own work is as rigorous and valid as possible [29]. And as Ifran and colleagues [28] also argue, HRI researchers should aspire to establish robust and reliable scientific standards for empirical HRI research. The fact that research rigour is receiving increasing attention in the domain of HRI will only benefit the field [30]. Furthermore, in a recent opinion piece, our group has further emphasized and provided concrete examples where empirical HRI and social robotics research can follow open science practices and focus on ensuring high reproducibility of research findings [31•].

## What Can Social Robotics Learn from the Social, Behavioural, and Cognitive Sciences?

In order to most appropriately and convincingly provide answers to what makes a robot social, research will clearly benefit from a broader variety of empirical disciplines to provide a complementary outlook. One field we would argue provides particularly rich opportunities for interdisciplinary collaboration with social robotics is cognitive neuroscience. Cognitive neuroscience is the study of the biological procedures that support cognition [32]. When cognitive neuroscience theory and methods are applied to HRI research, they allow us to probe how the human brain processes and reacts to robots, and these insights, in turn, can help facilitate further development of social robots [33]. Previous research in cognitive neuroscience has used social robots to address questions regarding attention (e.g. [34–37]), theory of mind (e.g. [38–41]), mind perception (e.g. [42–45]), intention attribution (e.g. [40, 46, 47]), and decision making (e.g. [48, 49]).

As an example of this bidirectional loop of cognitive neuroscience research informing robotic design, iCub, the ‘robot child’, is based on theories of developmental psychology and cognitive neuroscience [50, 51] and was developed as a testbed for the theory of embodied cognition. This theory describes the phenomenon of learning and development through the physical interaction with the world through a human(oid) body [51]. Like a child exploring its environment, iCub was designed to manipulate its surroundings, imitate its human partners, and communicate with them. iCub has been used in cognitive neuroscience studies to investigate whether humans perceive it as intentional and as an agent with a mind [52, 53•]. Across several studies, it has been shown that the degree to which participants perceive the robot as behaving intentionally is profoundly shaped by participants’ knowledge or beliefs about the robot [47, 54].

In addition to cognitive neuroscience, research from psychology relating to social cognition is also informing social robotics development, and vice versa. Social cognition can be defined as the processing, storing, and application of information about social beings and situations, and this discipline can help establish a role for cognitive processes during social interactions with social robots. Moreover, using social robots as research tools, we can learn more about ourselves as humans through a social-cognitive lens [33, 55]. Social concepts like trust (e.g. [56–59]), attachment (e.g. [60]), empathy (e.g. [61]), acceptance (e.g. [57, 62]), and disclosure (e.g. [42, 63–67]) with social robots are being studied. In addition, the use of social robots is growing in complex social contexts such as those found in education (e.g. [58, 59, 68]), service (e.g. [69]), and care sectors (e.g. [70–72]).

It is worth noting that several commercial robots that are widely used in research are strongly informed by (and continue to inform) social, behavioural, and cognitive science domains. Some of these robots take on a humanoid form, such as the Pepper and Nao robots by SoftBank Robotics (Figs. 1 and 2). Mubin and colleagues [73] investigated the use of Pepper and Nao in public spaces, and a range of studies have evaluated Pepper’s social acceptability in shopping malls, elderly care homes, remote classrooms, and as a customer service employee in a hotel lobby [24, 74–76]. While in these contexts a humanoid robot may be valuable, other developers have taken a different approach with the MiRo robot (Consequential Robotics). It is designed as a biomimetic system and its design (in terms of form and function) does not aim to be humanlike (Fig. 2), but instead takes its cues from (lower) mammalian brain and behavioural systems (such as a rabbit or dog [77]). The developers explicitly justify their design choice of animal morphology as a strategy to mitigate potential disappointment of users and their expectations towards the social capabilities of the robot. The design of the robot features light patterns under the translucent shell of the back, which satisfies two goals: the simple communication of affect and increasing the salience of the interaction with an artificial, rather than a real, social agent [77]. The robot, which evokes a pet-like impression, includes characteristics modelled from “puppies, kittens and rabbits” ([77], p. 2). This robot is described as an ‘edutainment’ product, which alludes to its intended purpose as an educational tool for children. However, MiRo has also been explored as a fall alert system, relevant especially to the elderly population [78]. In their proof-of-principle study, these authors demonstrated that MiRo could be used as a mobile and smart tool to locate a person on the ground, and send a help signal if no movement of the person is detected. These different embodiments for social robots highlight that in different contexts different types of social robots are valuable and appropriate.

To summarize, this section highlights how theoretical underpinnings and empirical work spanning the social, behavioural, and cognitive sciences can inform the development and deployment of social robots. While the field of social robotics seems to be in unanimous agreement that greater integration with these fields will accelerate and enhance social robotic development, challenges to working across disciplines remain (as discussed in the previous section), and will be important to overcome if the social robotics applications surveyed in the following section are to be introduced on a bigger scale. Continuing research with different types of social robot morphologies utilizing social sciences’ rigour and methodology will ultimately lead to an advancement in social robotics.

## Social Robots Deployed in the Wild

Recalling the cautionary tale of the Jibo robot introduced above, this story too has a happy ending. Earlier this year (March 2020), the assets for Jibo were acquired by the Japanese telecommunications company Nippon Telegraph and Telephone (NTT) [79]. Interestingly, NTT decided to focus Jibo’s future in health care and education. Instead of focusing on developing Jibo as a personal assistant robot that people can buy and use straight out of the box, NTT plans to market Jibo to businesses that provide certain services (such as healthcare and education) as a tool for professionals to use [80, 81]. Supporting this decision is NTT’s assessment that Jibo will be more valuable as an enterprise product in these designated domains, rather than as a consumer product. Surveying this area more broadly, the application of social robots within care settings, and as tools to deliver health and well-being interventions, is already an emerging success story highlighting contexts and uses where social robots are successfully being deployed as autonomous assistance tools for human users [82]. While it remains uncontroversial that social robots do not (yet) offer the same opportunities as humans for social interactions [33], they can nonetheless afford valuable opportunities for social engagement with human users when introduced in specific contexts, and in careful, ethically responsible ways [83, 84]. A growing evidence base documents how social robots might function as autonomous tools to support psychological health interventions [42, 85], physical therapy and physical health [86–88], and other means to amplify or support human therapeutic efforts (see [89•, 90]). Moreover, social robots are being equipped with technologies such as sensors, cameras, and processors, which promote the collection of human data (such as where a person is standing, where they are looking, what they are saying, etc.) with high fidelity, as well as support on-line, on-going analysis of a human interaction partner’s behaviour.

Research into the application of social robots in psychosocial health interventions highlights how social robots that take on different forms of embodiment and design can benefit different interventions. For example, robots like Paro, which take on a zoomorphic pet- or cuddly toy-like embodiment, hold value for interventions when used with appropriate target populations, including older adults in care homes and people with cognitive impairment (e.g. dementia) [91, 92]. A review by Hung and colleagues [93] found that previous studies using Paro provided evidence of this robot reducing negative emotions in patients, improving their social engagement, and generally promoting positive mood, atmosphere, and quality of care experience. Moreover, a recent study documents the psychophysiological benefits of interacting with a companion robot like Paro, demonstrating that stroking Paro reduces pain perception and salivary oxytocin levels [94]. Other research demonstrates how different robot forms can have negligible impact on psychosocial health interventions. A recent study by our group [42] examined how social robot and voice assistant technology might be used to support people’s psychological health through conversation. While participants were aware of many of the obvious differences between speaking to a humanoid social robot compared to a disembodied conversational agent (the Google Nest Mini voice assistant, in this case), their verbal disclosures to both were similar in length and duration. This finding thus suggests that human-like embodiment for this particular kind of conversational intervention did not lead to improved outcomes.

In contrast, health interventions where more active participation is required are finding that robots with a more human-like embodiment are more effective. One such study by da Silva and colleagues [95] tested an intervention for students with the humanoid Nao robot, aimed at encouraging their motivation to exercise through motivational interviewing. The results of their study demonstrated that some participants felt that the intervention increased their physical activity levels and their motivation to exercise. Interestingly, participants expressed a positive opinion of Nao as it appeared to be non-judgmental. This is a meaningful benefit of using social robots in psychosocial interventions, as these machines can overcome some of the social desirability limitations when similar interventions are operated exclusively by people. Another study that used Nao demonstrated its viability to deliver a behaviour change intervention, applying a motivational intervention for reducing high-calorie snack consumption [96]. This study reported a > 50% snack episode reduction between the beginning of the intervention and week 8, and an average weight reduction of 4.4 kg over the first 2 weeks of the treatment. Four weeks from the beginning of the intervention, participants reported an increase in their perceived confidence in controlling their snack intake and their emotional states. The results of this study demonstrate that in certain contexts and settings, social robots have potential to autonomously behaviour change interventions. While some evidence suggests that an intervention delivered by a social robot could be as effective as a human delivering a similar intervention (e.g. [96]), many significant open questions remain regarding the cost, ethics, and long-term efficacy of machine vs. human-based health interventions.

Social robots with more degrees of freedom in terms of their movement and behavioural repertoire can provide more advanced assistance, for example, by demonstrating complex physical movements to assist with rehabilitation, build physical fitness, and help people cope with injury and illness [88]. A recent study by Feingold-Polak and Levi-Tzedek [97] reported positive outcomes for a long-term upper limb rehabilitation intervention delivered via the humanoid social robot Pepper for post-stroke patients in a rehabilitation facility. Moreover, clinicians and patients in this study found the intervention with Pepper to be engaging, motivating, and most importantly meeting the needs of upper limb rehabilitation. Similar work has examined how the smaller, less expensive Nao robot can also deliver physical therapy for upper limb impairment, and shows similar effectiveness of this robot in rehabilitation contexts with adults [86]. Furthermore, Chen and colleagues [87] have shown that an even more compact and simple social robot (Darwin from RobotLab, San Francisco, CA, USA) can be effectively deployed to assist with children with and without cerebral palsy performing reach actions. This work further underscores the potential value and utility of embodied social robots for building physical capacity in individuals across the lifespan.

To summarize the state of the art on the potential of social robots to contribute to the greater good of society, increasing research effort is being invested in this domain, and some early results speaking to how robots might be able to support human psychosocial and physical function is promising. The current public health crisis has thrown into even starker contrast the value and need for not just technological solutions, but *embodied* technological solutions to help people stave off loneliness, as well as learn and connect with others when in-home learning and social distancing are the new normal [98]. Social robotics can undoubtedly contribute to improving people’s quality of life [99], but the need remains for more methodologically rigorous and ethically sound research into how social robots might interact with humans in a sensitive, timely and nuanced manner.

## Conclusions

In this review, we reflected on the paradox of robots’ limited socialness, and how it can be better defined, studied, and applied. It is apparent from the literature that a substantial gap remains between how social robots are defined by scientists and roboticists, compared to the general public’s expectations and experience with robots. Social robotics remains a small subdiscipline of robotics that envisions robots as assistants and companions. As this review highlights, it is also a heterogenous and multidisciplinary field, which can greatly benefit from deeper integration with and feedback from the social, behavioural, and neurocognitive sciences. The research reviewed here shows how, despite real limitations in social robots capabilities due to the current state of technology, they nonetheless hold potential to enhance human life, particularly in some education, psychosocial support, and rehabilitation contexts. The research reviewed in the context of these robots further highlights their usefulness as a testbed for human social cognition, in terms of probing its flexibility and dimensions [100]. Despite this, many questions remain regarding the capabilities of robots to take on more social roles, especially if they are to be working autonomously alongside human users in complex social settings.

## References

[1] Frumer Y. The short, strange life of the first friendly robot. IEEE SPECTRUM*.* 2020. https://spectrum.ieee.org/robotics/humanoids/the-short-strange-life-of-the-first-friendly-robot. Accessed 21 May 2020.

[2] Schwartz O. Untold history of AI: when charles babbage played chess with the original mechanical Turk. IEEE SPECTRUM. 2019. Available in https://spectrum.ieee.org/tech-talk/tech-history/dawn-of-electronics/untold-history-of-ai-charles-babbage-and-the-turk. Accessed 18 Mar 2019.

[3] Broadbent E (2017). Interactions with robots: the truths we reveal about ourselves. Annu Rev Psychol.

[4] Duffy BR, Joue G. The paradox of social robotics: a discussion. AAAI Fall 2005 Symp Mach ethics. Hyatt Regency; 2005.

[5] Pandey AK, Gelin R (2018). A mass-produced sociable humanoid robot: pepper: the first machine of its kind. IEEE Robot Autom Mag.

[6] de Graaf MMA, Ben Allouch S, van Dijk JAGM, Tapus A, André E, Martin J-C, Ferland F, Ammi M (2015). What makes robots social?: a user’s perspective on characteristics for social human-robot interaction. Soc robot.

[7] Dereshev D, Kirk D, Matsumura K, Maeda T (2019). Long-term value of social robots through the eyes of expert users. Proc 2019 CHI Conf Hum Factors Comput Syst.

[8] Rivoire C, Lim A (2016). Habit detection within a long-term interaction with a social robot: an exploratory study. Proc Int Work Soc Learn Multimodal Interact Des Artif Agents.

[9] Breazeal C. JIBO, The world’s first social robot for the home [Internet]. Indiegogo. 2014. Available from: https://www.indiegogo.com/projects/jibo-the-world-s-first-social-robot-for-the-home#/. Accessed 15 Sep 2014.

[10] Hodson H (2014). The first family robot. New Sci.

[11] Martin D. Layoffs hit Jibo more than a month after social robot’s launch [Internet]. BostInno. 2017. Available from: https://www.bizjournals.com/boston/inno/stories/news/2017/12/15/layoffs-hit-jibo-more-than-a-month-after-social.html. Accessed 15 Dec 2017.

[12] Ackerman E. Jibo is probably totally dead now - [Internet]. IEEE Spectr. 2018. Available from: https://spectrum.ieee.org/automaton/robotics/home-robots/jibo-is-probably-totally-dead-now. Accessed 3 Dec 2018.

[13] Heater B. The lonely death of Jibo, the social robot [Internet]. TechCrunch. 2019. Available from: https://techcrunch.com/2019/03/04/the-lonely-death-of-jibo-the-social-robot/?guccounter=1&guce_referrer=aHR0cHM6Ly93d3cuZ29vZ2xlLmNvbS8&guce_referrer_sig=AQAAAKxruRDCPEMeI3RcsWrOES7hl3N5odhwrQH8w4HTsSHkaduBw8aaiaYgmVURrvZATXJAvQ5FM5NXpCK8ih5ERQ1gwF9jHxX1X36hQVABezUeFrg9BCbbkNUwmYay3vBxVd5hMrLTodPu6PrYK47oAHWLMzxUa6waS-1SBtT1l2eo. Accessed 4 Mar 2019.

[14] Kinsella B. Consumer robots are dead; long live Alexa [Internet]. USA Today Tech. 2018. Available from: https://eu.usatoday.com/story/tech/talkingtech/2018/12/13/consumer-robots-dead-long-live-alexa/2272460002/. Accessed 13 Dec 2018.

[15] Kinsella B. Jibo Shuts down, selling off robot parts [Internet]. Voicebot.ai. 2018. Available from: https://voicebot.ai/2018/12/03/jibo-shuts-down-selling-off-robot-parts/. Accessed 3 Dec 2018.

[16] Linus Tech Tips. TERRIBLE $900 party trick – Jibo review [Video file] [Internet]. 2017. Available from: https://www.youtube.com/watch?v=U1RASlbIIVc&feature=youtu.be. Accessed 27 Dec 2017.

[17] Williams A. Virtual assistants evolve, but will they be integrated in robots? [Internet]. Robot. Bus. Rev. 2018. Available from: https://www.roboticsbusinessreview.com/consumer/virtual-assistants-integrated-robots/. Accessed 8 Oct 2018.

[18] Goodrich MA, Schultz AC (2007). Human-robot interaction: a survey. found trends hum-comput interact.

[19] Mejia C, Kajikawa Y (2017). Bibliometric analysis of social robotics research: identifying research trends and knowledgebase. Appl Sci.

[20] Šabanović S (2010). Robots in society, society in robots: mutual shaping of society and technology as a framework for social robot design. Int J Soc Robot.

[21] Sarrica M, Brondi S, Fortunati L (2019). How many facets does a “social robot” have? A review of scientific and popular definitions online. Inf Technol People.

[22] Dautenhahn K (2007). Socially intelligent robots: dimensions of human-robot interaction. Philos Trans R Soc Lond B Biol Sci The Royal Soc.

[23] Leite I, Martinho C, Paiva A (2013). Social robots for long-term interaction: a survey. Int J Soc Robot.

[24] Tanaka F, Isshiki K, Takahashi F, Uekusa M, Sei R, Hayashi K. Pepper learns together with children: development of an educational application. 2015 IEEE-RAS 15th International Conference on Humanoid Robots (Humanoids). 2015:270–5. 10.1109/HUMANOIDS.2015.7363546.

[25] Baraka K, Alves-Oliveira P, Ribeiro T. An extended framework for characterizing social robots. In: Jost C, Le Pévédic B, Belpaeme T, Bethel C, Chrysostomou D, Crook N, et al., editors. Human-Robot Interact Eval Methods Their Stand Springer Ser Bio- Neurosystems: Springer International Publishing; 2020. p. 21–64. 10.1007/978-3-030-42307-0_2.

[26] Cross ES, Ramsey R. Mind meets machine: towards a cognitive science of human-machine interactions. Trends Cogn Sci. 2020;28:S1364–6613(20)30297–7. 10.1016/j.tics.2020.11.009. 10.1016/j.tics.2020.11.009 33384213

[27] Eyssel F (2017). An experimental psychological perspective on social robotics. Robot Auton Syst.

[28] Irfan B, Kennedy J, Lemaignan S, Papadopoulos F, Senft E, Belpaeme T. Social psychology and human-robot interaction: an uneasy marriage. Companion 2018 ACM/IEEE Int Conf Human-Robot Interact, vol. 2018. New York: Association for Computing Machinery. p. 13–20. 10.1145/3173386.3173389.

[29] Belpaeme T. Learning from social robots. 2020 Int Symp Community-centric Syst. Hachioji, Tokyo, Japan; 2020. p. 12. 10.1109/CcS49175.2020.9231310.

[30] Hoffman G, Zhao X (2020). A primer for conducting experiments in human–robot interaction. J Hum-Robot Interact.

[31] Henschel A, Hortensius R, Cross ES (2020). Social cognition in the age of human–robot interaction. Trends Neurosci.

[32] Gazzaniga MS, Mangun GR (2014). The cognitive neurosciences.

[33] Cross ES, Hortensius R, Wykowska A (2019). From social brains to social robots: applying neurocognitive insights to human-robot interaction. Philos Trans R Soc B Biol Sci.

[34] Cao W, Song W, Li X, Zheng S, Zhang G, Wu Y (2019). Interaction with social robots: Improving gaze toward face but not necessarily joint attention in children with autism spectrum disorder. Front Psychol.

[35] Chevalier P, Kompatsiari K, Ciardo F, Wykowska A (2020). Examining joint attention with the use of humanoid robots-a new approach to study fundamental mechanisms of social cognition. Psychon Bull Rev.

[36] Gordon G (2019). Social behaviour as an emergent property of embodied curiosity: a robotics perspective. Philos Trans R Soc B Biol Sci.

[37] Kajopoulos J, Cheng G, Kise K, Müller HJ, Wykowska A (2020). Focusing on the face or getting distracted by social signals? The effect of distracting gestures on attentional focus in natural interaction. Psychol Res.

[38] Banks J (2020). Theory of mind in social robots: replication of five established human tests. Int J Soc Robot.

[39] Bianco F, Ognibene D, Salichs MA, Ge SS, Barakova EI, Cabibihan J-J, Wagner AR, Castro-González Á (2019). Transferring adaptive theory of mind to social robots: insights from developmental psychology to robotics.

[40] Bossi F, Willemse C, Cavazza J, Marchesi S, Murino V, Wykowska A (2020). The human brain reveals resting state activity patterns that are predictive of biases in attitudes toward robots. Sci Robot.

[41] Kuniyoshi Y (2019). Fusing autonomy and sociality via embodied emergence and development of behaviour and cognition from fetal period. Philos Trans R Soc B Biol Sci.

[42] Laban G, George J-N, Morrison V, Cross E. Tell me more! Assessing interactions with social robots from speech. Paladyn J Behav Robot. 2021;12:136–159. 10.1515/pjbr-2021-0011.

[43] Stafford RQ, MacDonald BA, Jayawardena C, Wegner DM, Broadbent E (2014). Does the robot have a mind? Mind perception and attitudes towards robots predict use of an eldercare robot. Int J Soc Robot.

[44] Wallkötter S, Stower R, Kappas A, Castellano G. A robot by any other frame: framing and behaviour influence mind perception in virtual but not real-world environments. Proc 2020 ACM/IEEE Int Conf human-robot interact, vol. 2020. New York: Association for Computing Machinery. p. 609–18. 10.1145/3319502.3374800.

[45] Wang X, Krumhuber EG (2018). Mind perception of robots varies with their economic versus social function. Front Psychol.

[46] Thellman S, Ziemke T (2020). *Do* you see what i see? Tracking the perceptual beliefs of robots. iScience..

[47] Wiese E, Metta G, Wykowska A (2017). Robots as intentional agents: using neuroscientific methods to make robots appear more social. Front Psychol.

[48] Hsieh T-Y, Chaudhury B, Cross ES. Human-robot cooperation in prisoner dilemma games: people behave more reciprocally than prosocially toward robots. Companion 2020 ACM/IEEE Int Conf human-robot interact, vol. 2020. New York: Association for Computing Machinery. p. 257–9. 10.1145/3371382.3378309.

[49] Marchesi S, Perez-Osorio J, De Tommaso D, Wykowska A (2020). Don’t overthink: fast decision making combined with behavior variability perceived as more human-like. 2020 29th IEEE Int Conf Robot Hum Interact Commun.

[50] Natale L, Bartolozzi C, Pucci D, Wykowska A, Metta G (2017). iCub: the not-yet-finished story of building a robot child. Sci Robot.

[51] Sandini G, Metta G, Vernon D. RobotCub: an open framework for research in embodied cognition. 4th IEEE/RAS Int Conf Humanoid Robot 2004. 2004; Vol. 1. p. 13–32 10.1109/ICHR.2004.1442111.

[52] Ghiglino D, De Tommaso D, Willemse C, Marchesi S, Wykowska A. Can I get your (robot) attention? Human sensitivity to subtle hints of human-likeness in a humanoid robot’s behavior. 2020. 10.31234/osf.io/kfy4g.

[53] • Pérez-Osorio J, De Tommaso D, Baykara E, Wykowska A. Joint action with Icub: a successful adaptation of a paradigm of cognitive neuroscience in HRI. 2018 27th IEEE Int Symp Robot Hum Interact Commun. 2018:152–7. 10.1109/ROMAN.2018.8525536**This seminal work highlights the cutting-edge research performed by Wykowska and colleagues at the IIT develop cognitive neuroscience paradigms with the iCub robot, which enable research into fundamental aspects of social perception and cognition (such as joint attention) using real-life social scenarios with embodied robots.**

[54] Wykowska A, Chaminade T, Cheng G (2016). Embodied artificial agents for understanding human social cognition. Philos Trans R Soc B Biol Sci.

[55] Wykowska A. Social robots to test flexibility of human social cognition. Int J Soc Robot. 2020:1–9. 10.1007/s12369-020-00674-5. 10.1007/s12369-020-00674-5 PMC7773613 33408797

[56] Langer A, Feingold-Polak R, Mueller O, Kellmeyer P, Levy-Tzedek S (2019). Trust in socially assistive robots: considerations for use in rehabilitation. Neurosci Biobehav Rev.

[57] Naneva S, Sarda Gou M, Webb TL, Prescott TJ. A systematic review of attitudes, anxiety, acceptance, and trust towards social robots. Int J Soc Robot. 2020:1–23. 10.1007/s12369-020-00659-4.

[58] Stower R, Kappas A. “Oh no, my instructions were wrong!” An exploratory pilot towards children’s trust in social robots. 2020 29th IEEE Int Conf robot hum interact Commun. Naples, Italy; 2020. p. 641–6. 10.1109/RO-MAN47096.2020.9223495.

[59] Stower R. The Role of trust and social behaviours in children’s learning from social robots. 2019 8th Int Conf Affect Comput Intell Interact Work Demos. Cambridge, United Kingdom, 2019. p. 1–5. 10.1109/ACIIW.2019.8925269.

[60] Dziergwa M, Kaczmarek M, Kaczmarek P, Kędzierski J, Wadas-Szydłowska K (2018). Long-term cohabitation with a social robot: a case study of the influence of human attachment patterns. Int J Soc Robot.

[61] Cross ES, Riddoch KA, Pratts J, Titone S, Chaudhury B, Hortensius R (2019). A neurocognitive investigation of the impact of socializing with a robot on empathy for pain. Philos Trans R Soc B Biol Sci.

[62] Thunberg S, Thellman S, Ziemke T (2017). Don’t judge a book by its cover: a study of the social acceptance of NAO vs. Pepper. Proc 5th Int Conf Hum Agent Interact.

[63] Birnbaum GE, Mizrahi M, Hoffman G, Reis HT, Finkel EJ, Sass O (2016). What robots can teach us about intimacy: the reassuring effects of robot responsiveness to human disclosure. Comput Hum Behav.

[64] Birnbaum GE, Mizrahi M, Hoffman G, Reis HT, Finkel EJ, Sass O. Machines as a source of consolation: robot responsiveness increases human approach behavior and desire for companionship. 2016 11th ACM/IEEE Int Conf Human-Robot Interact. 2016. p. 165–72. 10.1109/HRI.2016.7451748.

[65] Björling EA, Rose E, Davidson A, Ren R, Wong D (2019). Can we keep him forever? Teen’s engagement and desire for emotional connection with a social robot. Int J Soc Robot.

[66] Hoffman G, Birnbaum GE, Vanunu K, Sass O, Reis HT. Robot Responsiveness to human disclosure affects social impression and appeal. Proc 2014 ACM/IEEE Int Conf Human-Robot Interact, vol. 2014. New York: Association for Computing Machinery. p. 1–8. 10.1145/2559636.2559660.

[67] Traeger ML, Sebo SS, Jung M, Scassellati B, Christakis NA (2020). Vulnerable robots positively shape human conversational dynamics in a human–robot team. Proc Natl Acad Sci.

[68] Belpaeme T, Kennedy J, Ramachandran A, Scassellati B, Tanaka F (2018). Social robots for education: a review. Sci Robot.

[69] Čaić M, Mahr D, Oderkerken-Schröder G (2019). Value of social robots in services: social cognition perspective. J Serv Mark.

[70] Dawe J, Sutherland C, Barco A, Broadbent E (2019). Can social robots help children in healthcare contexts? A scoping review. BMJ Paediatr Open.

[71] Johanson DL, Ahn HS, MacDonald BA, Ahn BK, Lim J, Hwang E (2019). The effect of robot attentional behaviors on user perceptions and behaviors in a simulated health care interaction: randomized controlled trial. J Med Internet Res.

[72] Johanson DL, Ho SA, Sutherland CJ, Brown B, MacDonald BA, Jong YL (2020). Smiling and use of first-name by a healthcare receptionist robot: effects on user perceptions, attitudes, and behaviours. Paladyn J Behav Robot.

[73] Mubin O, Ahmad MI, Kaur S, Shi W, Khan A, Ge SS, Cabibihan J-J, Salichs MA, Broadbent E, He H, Wagner AR (2018). Social robots in public spaces: a meta-review. Soc Robot.

[74] Aaltonen I, Arvola A, Heikkilä P, Lammi H (2017). Hello Pepper, may i tickle you? Children’s and adults’ responses to an entertainment robot at a shopping mall. Proc Companion 2017 ACM/IEEE Int Conf Human-Robot Interact.

[75] Stock R. M., Merkle M. Can humanoid service robots perform better than service employees? A comparison of innovative behavior cues. Proceedings of the 51st Hawaii International Conference on System Sciences 2018:10.

[76] Yang C, Lu M, Tseng S, Fu L. A companion robot for daily care of elders based on homeostasis. 2017 56th Annu Conf Soc Instrum Control Eng Japan. 2017. p. 1401–6. 10.23919/SICE.2017.8105748.

[77] Collins EC, Prescott TJ, Mitchinson B, Conran S (2015). MIRO: a versatile biomimetic edutainment robot. Proc 12th Int Conf Adv Comput Entertain Technol.

[78] Georgiou T, Singh K, Baillie L, Broz F (2020). Small robots with big tasks: a proof of concept implementation using a MiRo for fall alert. companion 2020 ACM/IEEE Int Confuman-robot interact.

[79] Crowe S. Jibo’s social robot assets acquired by NTT disruption [Internet]. Robot Rep. 2020. Available from: https://www.therobotreport.com/jibosocial-robot-assets-acquired-ntt-disruption/. Accessed 18 Mar 2020.

[80] Carman A. Jibo, the social robot that was supposed to die, is getting a second life. The Verge 2020. Available from: https://www.theverge.com/2020/7/23/21325644/jibo-social-robot-ntt-disruptionfunding. Accessed 23 July 2020.

[81] NTT Disruption. Jibo the social robot returns, with its brand new website - NTT DISRUPTION | Creating today what really matters for tomorrow. 2020. Available from: https://disruption.global.ntt/jibo-the-social-robot-returns-with-its-brand-new-website/. Accessed 23 July 2020.

[82] Cifuentes CA, Pinto MJ, Céspedes N, Múnera M (2020). Social robots in therapy and care. Curr Robot Rep.

[83] Villaronga EF, Kieseberg P, Li T (2018). Humans forget, machines remember: artificial intelligence and the right to be forgotten. Comput Law Secur Rev.

[84] Wullenkord R, Eyssel F (2020). Societal and ethical issues in HRI. Curr Robot Rep.

[85] Alnajjar F, Khalid S, Vogan AA, Shimoda S, Nouchi R, Kawashima R (2019). Emerging cognitive intervention technologies to meet the needs of an aging population: a systematic review. Front Aging Neurosci.

[86] Assad-Uz-Zaman M, Rasedul Islam M, Miah S, Rahman MH (2019). NAO robot for cooperative rehabilitation training. J Rehabil Assist Technol Eng.

[87] Chen Y, Garcia-Vergara S, Howard AM (2018). Effect of feedback from a socially interactive humanoid robot on reaching kinematics in children with and without cerebral palsy: a pilot study. Dev Neurorehabil.

[88] Mohebbi A (2020). Human-robot interaction in rehabilitation and assistance: a review. Curr Robot Rep.

[89] Robinson NL, Cottier TV, Kavanagh DJ (2019). Psychosocial health interventions by social robots: systematic review of randomized controlled trials. J Med Internet Res.

[90] Scoglio AAJ, Reilly ED, Gorman JA, Drebing CE (2019). Use of social robots in mental health and well-being research: systematic review. J Med Internet Res.

[91] Góngora Alonso S, Hamrioui S, de la Torre DI, Motta Cruz E, López-Coronado M, Franco M (2018). Social robots for people with aging and dementia: a systematic review of literature. Telemed e-Health.

[92] Robinson H, MacDonald B, Kerse N, Broadbent E (2013). The psychosocial effects of a companion robot: a randomized controlled trial. J Am Med Dir Assoc.

[93] Hung L, Liu C, Woldum E, Au-Yeung A, Berndt A, Wallsworth C, Horne N, Gregorio M, Mann J, Chaudhury H (2019). The benefits of and barriers to using a social robot PARO in care settings: a scoping review. BMC Geriatr.

[94] Geva N, Uzefovsky F, Levy-Tzedek S (2020). Touching the social robot PARO reduces pain perception and salivary oxytocin levels. Sci Rep.

[95] da Silva J, Kavanagh DJ, Belpaeme T, Taylor L, Beeson K, Andrade J (2018). Experiences of a motivational interview delivered by a robot: qualitative study. J Med Internet Res.

[96] Robinson NL, Connolly J, Hides L, Kavanagh DJ (2020). Social robots as treatment agents: pilot randomized controlled trial to deliver a behavior change intervention. Internet Interv.

[97] Feingold Polak R, Tzedek SL. Social robot for rehabilitation: expert clinicians and post-stroke patients’ evaluation following a long-term intervention. Proc 2020 ACM/IEEE Int Conf Human-Robot Interact, vol. 2020. New York: Association for Computing Machinery. p. 151–60. 10.1145/3319502.3374797.

[98] Henschel A, Cross ES. The neuroscience of loneliness – and how technology is helping us [Internet]. Conversat. 2020. Available from: https://theconversation.com/the-neuroscience-of-loneliness-and-how-technology-is-helping-us-136093. Accessed 17 Apr 2020.

[99] Yang G-Z, Nelson BJ, Murphy RR, Choset H, Christensen H, Collins SH (2020). Combating COVID-19—the role of robotics in managing public health and infectious diseases. Sci Robot.

[100] Hortensius R, Cross ES (2018). From automata to animate beings: the scope and limits of attributing socialness to artificial agents. Ann N Y Acad Sci.

